# Calcium-Sensing Receptor in Adipose Tissue: Possible Association with Obesity-Related Elevated Autophagy

**DOI:** 10.3390/ijms21207617

**Published:** 2020-10-15

**Authors:** Pamela Mattar, Sofía Sanhueza, Gabriela Yuri, Lautaro Briones, Claudio Perez-Leighton, Assaf Rudich, Sergio Lavandero, Mariana Cifuentes

**Affiliations:** 1 Institute of Nutrition and Food Technology (INTA), University of Chile, Santiago 7830490, Chile; pmattar@inta.uchile.cl (P.M.); sofiasanhuezag@gmail.com (S.S.); gyuri@uc.cl (G.Y.); lbriones@ubiobio.cl (L.B.); 2 Department of Nutrition and Public Health, Faculty of Health Sciences, University of Bío-Bío, Andrés Bello 720, Chillán 3800708, Chile; 3 Department of Physiology, Faculty of Biological Sciences, Pontifical Catholic University of Chile, Santiago 3580000, Chile; cperezl@bio.puc.cl; 4 Department of Clinical Biochemistry and Pharmacology, Ben-Gurion University of the Negev, Beer-Sheva 84103, Israel; rudich@bgu.ac.il; 5 The National Institute of Biotechnology in the Negev, Ben-Gurion University of the Negev, Beer-Sheva 84103, Israel; 6 Advanced Center for Chronic Diseases (ACCDiS), Faculty of Chemical and Pharmaceutical Sciences & Faculty of Medicine, University of Chile, Santiago 8380492, Chile; slavander@uchile.cl; 7 Center for Exercise, Metabolism and Cancer (CEMC), Faculty of Medicine, University of Chile, Santiago 8380492, Chile; 8 Department of Internal Medicine, Cardiology Division, University of Texas Southwestern Medical Center, Dallas, TX 75390, USA

**Keywords:** calcium-sensing receptor, autophagy, visceral adipose tissue, obesity

## Abstract

Autophagy is upregulated in adipose tissue (AT) from people with obesity. We showed that activation of the calcium-sensing receptor (CaSR) elevates proinflammatory cytokines through autophagy in preadipocytes. Our aim is to understand the role of CaSR on autophagy in AT from humans with obesity. We determined mRNA and protein levels of CaSR and markers of autophagy by qPCR and western blot in human visceral AT explants or isolated primary preadipocytes (60 donors: 72% female, 23–56% body fat). We also investigated their association with donors’ anthropometric variables. Donors’ % body fat and CaSR mRNA expression in AT were correlated (r = 0.44, *p* < 0.01). CaSR expression was associated with mRNA levels of the autophagy markers *atg5* (r = 0.37, *p* < 0.01), *atg7* (r = 0.29, *p* < 0.05) and *lc3b* (r = 0.40, *p* < 0.01). CaSR activation increased *becn* and *atg7* mRNA expression in AT. CaSR activation also upregulated LC3II by ~50%, an effect abolished by the CaSR inhibitor. Spermine (CaSR agonist) regulates LC3II through the ERK1/2 pathway. Structural equation model analysis suggests a link between donors’ AT CaSR expression, AT autophagy and expression of Tumor Necrosis Factor alpha TNF-α. CaSR expression in visceral AT is directly associated with % body fat, and CaSR activation may contribute to obesity-related disruption in AT autophagy.

## 1. Introduction

Autophagy is a conserved intracellular catabolic mechanism that delivers damaged organelles and macromolecules to lysosomes for degradation, which is critical for energy supply and cellular homeostasis. In vivo and in vitro studies have proposed that autophagy is dysregulated in obesity in a tissue/cell-dependent manner [1,2,3,4], contributing to development of cardiometabolic comorbidities. In the liver and pancreas, obesity-associated decreased autophagy has been linked to development of fatty liver [5] and type 2 diabetes T2D [6]. In contrast, adipose tissue (AT) from patients with obesity and T2D shows enhanced autophagy [1,2,7]. A study in AT from patients with obesity showed greater mRNA expression of the autophagy-related genes *Atg5*, *lc3a* and *lc3b* than their lean counterparts, as well as in visceral versus subcutaneous depot [2]. Interestingly, *Atg5*, *lc3a* and *lc3b* mRNA levels were higher in visceral AT of patients with obesity and insulin resistance as compared with those with obesity but who are insulin-sensitive [2], suggesting an association between increased AT autophagy markers and the presence of obesity-related metabolic derangements.

We described the presence of the G protein-coupled extracellular calcium-sensing receptor (CaSR) in human AT, and we proposed that this receptor may be involved in AT dysfunction [8]. CaSR was originally described for its key role in parathyroid hormone secretion and circulating calcium homeostasis, and subsequent research has shown its relevance in inflammation-related disorders [9,10], among others. CaSR can be activated by several ligands, including cations, antibiotics, polyamines and pharmacological compounds [11]. Multiple intracellular pathways can be triggered depending on the receptor’s conformational state induced by a given CaSR ligand, a phenomenon called biased agonism [11,12]. In preadipocytes, the allosteric CaSR activator cinacalcet induces ERK1/2 activation, resulting in cell proliferation and NLRP3 induction [13,14]. CaSR activation by the polyamine spermine and/or the calcimimetic cinacalcet in preadipocytes, adipocytes and whole adipose tissue is associated with inflammation, adipogenesis, proliferation, as well as impaired lipid handling [3,13,14,15,16,17].

Liu et al. observed in vitro that CaSR inhibition reduces cardiac hypertrophy by decreasing autophagy [18,19]. Several studies have confirmed that CaSR activation increases autophagy in THP-1 macrophages [20], cardiac fibroblasts [21] and kidney cells [22]. We recently reported that cinacalcet elevates autophagy in human preadipocytes [3], which may be part of the mechanistic scaffold involved in inflammation-linked AT dysfunction that induces obesity-related diseases.

Our previous work showed that CaSR activation in preadipocytes contributes to Tumor Necrosis Factor alpha TNF-α secretion by upregulating autophagy [3]. In the present work, we focus on human visceral AT explants to study whether CaSR expression is associated with obesity and if its activation induces autophagy in the tissue context. Our results show that CaSR expression is associated with donors’ body fat percentage (%BF), and that CaSR activation modulates autophagy-related mRNA transcripts and proteins, suggesting that CaSR may strengthen obesity-dysregulated autophagy in AT.

## 2. Results

### 2.1. CaSR mRNA Expression in Visceral Adipose Tissue is Positively Correlated with Fat Percentage and Autophagy Markers

**Figure 1:**
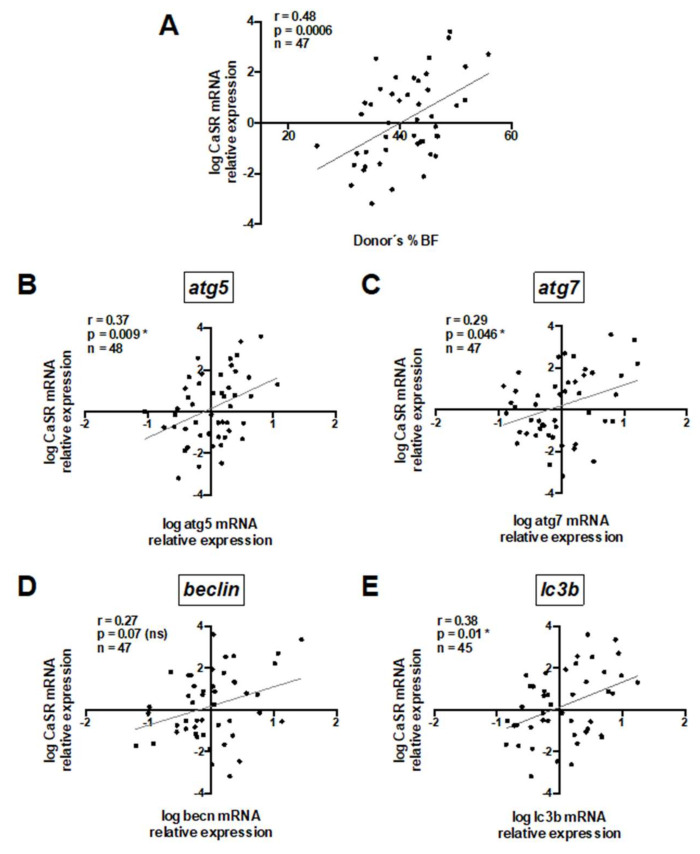
Whole adipose tissue calcium-sensing receptor (CaSR) mRNA content positively correlates with donors’ body fat percentage and mRNA of autophagy markers. Pearson’s correlation coefficient was calculated for the association between CaSR mRNA and (**A**) body fat percentage (%BF), (**B**) *atg5*, (**C**) *atg7*, (**D**) *becn* and (**E**) *lc3b*. All mRNA values were log transformed. Each graph depicts the r, and *p* values, as well as the number of explants analyzed (1 explant = 1 donor) (n). * Significant and ns = non-significant.

To understand the role of CaSR in human visceral AT, we firstly assessed CaSR mRNA expression in untreated whole visceral adipose tissue and evaluated its association with donors’ anthropometric variables and autophagy mRNA transcripts (Table S1). A significant positive correlation between CaSR mRNA expression and donors’ %BF is shown in Figure 1A. A trend towards a positive association between CaSR mRNA expression and BMI was observed (Table S1). In addition, there was a positive correlation between CaSR and mRNA transcripts of the autophagy markers *atg5*, *atg7* and *lc3b* (Figure 1B,C,E), and a trend (*p* = 0.07) for the same association with *beclin* (*becn*) (Figure 1D).

### 2.2. Cinacalcet and Spermine Increase Autophagy Markers in Visceral AT Explants

**Figure 2:**
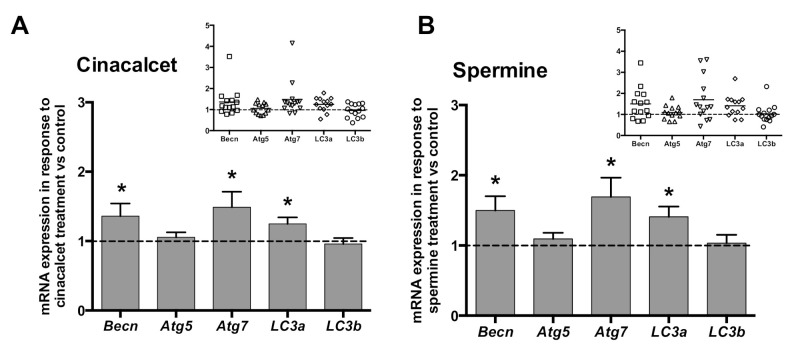
Expression (mRNA) of the autophagy genes *becn*, *atg7* and *lc3a* is upregulated by CaSR activators in whole adipose tissue explants. mRNA content was determined by qPCR after 6 h treatment with (**A**) 2 μM cinacalcet or (**B**) 100 μM spermine. mRNA expression is expressed normalized by the internal reference gene *GAPDH* and presented as a fold of vehicle-treated controls (dotted line, 1). * *p* < 0.05 for the difference from 1, Wilcoxon signed rank test, n = 13 independent donors’ explants. The insets show the individual experimental data.

**Figure 3:**
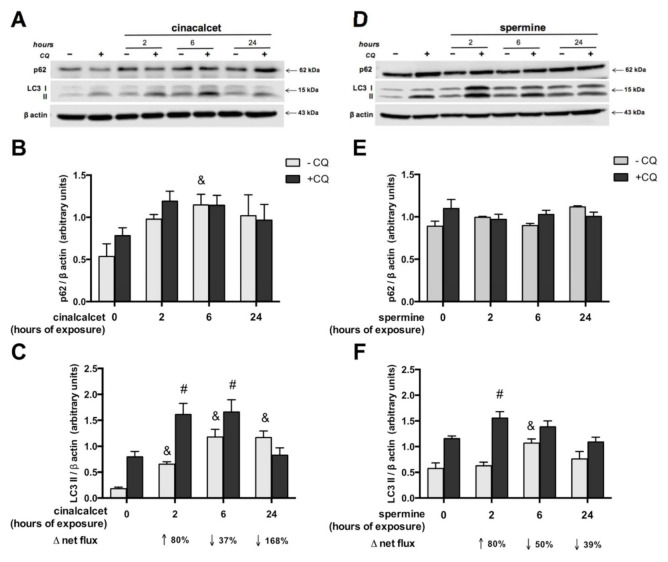
Autophagy flux is modulated by cinacalcet or spermine in human adipose tissue explants. LC3II and p62 protein expression was determined after 2, 6 and 24 h of (**A**–**C**) 2 μM cinacalcet or (**D**–**F**) 100 μM spermine with or without chloroquine (CQ) to determine the protein levels under autophagy flux inhibition or basal conditions, respectively. LC3 and p62 abundance was normalized by β-actin. Differences between vehicle and treated conditions were determined by two-way ANOVA and Holm–Sidak’s multiple comparison post-hoc test. The symbols “&” (−CQ) and “#” (+CQ) represent *p* < 0.05 compared to the respective vehicle condition, n = 4–6 independent experiments.

In order to investigate whether CaSR activators elevate visceral AT autophagy marker mRNA, AT explants were exposed to 2 μmol/L cincacalcet or 100 μmol/L spermine for 6 h, and mRNA transcripts were evaluated in whole adipose tissue by qPCR. Notably, transcriptional regulation is considered a relevant source of modulation of autophagy process, particularly in obesity-related AT dysfunction [23,24]. As shown in Figure 2A,B, exposure to either of the CaSR activators showed higher *becn*, a*tg7* and *lc3a* mRNA. Additionally, we evaluated post-transcriptional regulation by autophagy flux in response to cinacalcet or spermine, using the autophagosome degradation inhibitor chloroquine (CQ) to visualize the resulting net abundance of LC3II and p62. Autophagy flux was calculated by the difference in abundance of the marker (LC3II or p62) in conditions with CQ vs. without CQ [25]. Basal levels of p62 increased in response to 6h cinacalcet treatment (Figure 3A); however, there were no changes in p62 with CQ or in response to spermine (Figure 3A,C). Basal LC3II increased after 6 h of exposure to the activators (Figure 3C,F), with cinacalcet elevating the process at 2 h and remaining high at 24 h. Cinacalcet and spermine increased LC3II expression in the presence of CQ after 2 h, with the effect of cinacalcet remaining at 6 h (Figure 3C,F). Cinacalcet induced a non-significant trend (*p* = 0.1) to decrease autophagy flux measured by LC3II in a time-dependent manner (Figure 3C and Figure S1A), with a similar response for p62 (Figure S1B). These cinacalcet-driven changes in autophagy flux suggest a trend to increase autophagosome formation at 2 h, with a subsequent decrease in lysosomal degradation (at 24 h). In the case of spermine treatment, both LC3II and p62 show less consistent results for autophagy flux (Figure 3E,F and Figure S1C,D).

**Figure 4:**
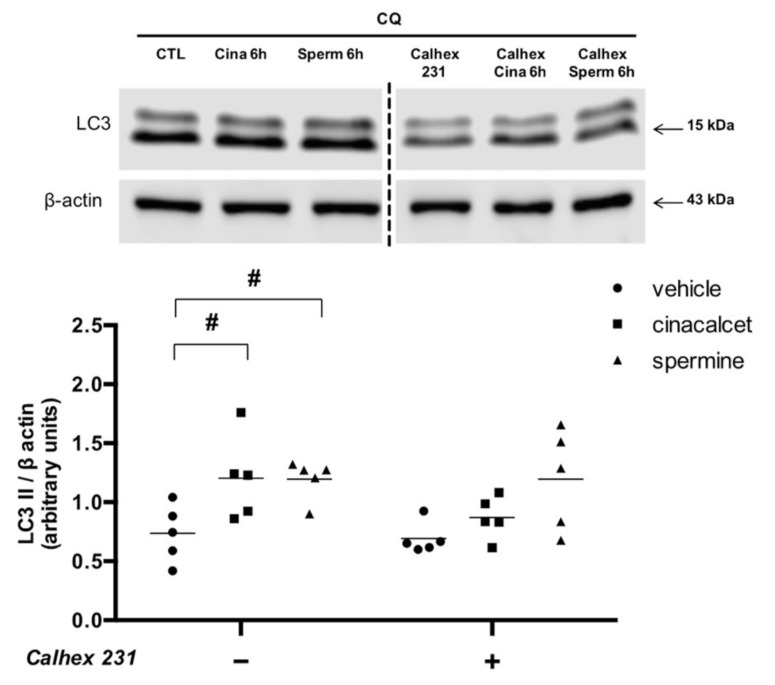
Cinacalcet- and spermine-induced elevation in autophagy is abolished by CaSR inhibition in visceral adipose tissue explants. Protein expression was determined after 6 h exposure to 2 μM cinacalcet or 100 μM spermine with or without 10 μM calhex 231 preincubation (1 h) and presence thereafter. All conditions were exposed to 20 μM chloroquine (CQ) during the last 2–3 h to determine the protein levels under autophagy flux inhibition. Expression of protein abundance was normalized by β-actin. Differences between vehicle and treated conditions were determined by two-way ANOVA and Holm–Sidak’s multiple comparison post-hoc test. ^#^ *p* < 0.05 and the dots represent each independent experiment (n = 5), corresponding to a different adipose tissue (AT) explant donor. The two parts of the membrane represent the same experiment and were processed in a single western blot.

To confirm that the effects of cinacalcet and spermine are mediated by CaSR activation, we evaluated changes in LC3II levels after preincubation with the CaSR inhibitor calhex 231. Figure 4 shows that pre-exposure to the inhibitor abolished the increase in LC3II induced by cinacalcet and spermine.

### 2.3. Spermine Regulates Autophagy through ERK1/2 Pathway in Primary Preadipocytes

**Figure 5:**
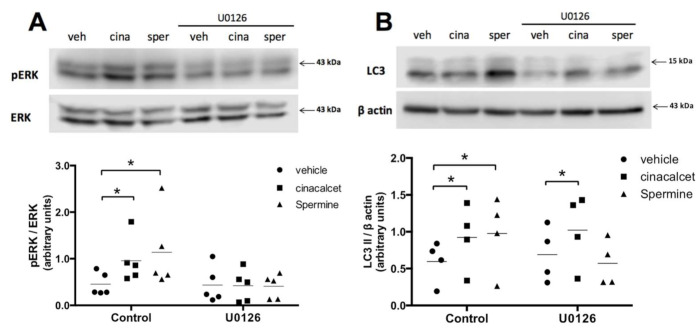
CaSR activation by spermine elevates autophagy through ERK1/2 in primary preadipocytes. (**A**) pERK/ERK and (**B**) LC3II levels were evaluated with or without pre-exposure to the upstream (MAPK) ERK1/2 phosphorylation inhibitor U0126 (10 μM) in primary isolated human preadipocytes treated with the CaSR activators cinacalcet (2 μM) or spermine (100 μM) or vehicle. The images show representative blots for each condition, and graphs summarize the densitometry analysis. Bars represent mean ± SEM, * *p* < 0.05 for two-way ANOVA versus vehicle or vehicle + U0126 and Holm–Sidak’s multiple comparison post-hoc test, n = 4–5.

To explore the mechanism through which CaSR modulates autophagy, we evaluated the ERK 1/2 pathway, given our previous reports of the relevance of this pathway in CaSR signaling in the human LS14 preadipocyte cell line [13,14]. We performed control experiments to assess ERK1/2 inhibition with the upstream (MEK1) ERK inhibitor U0126 in whole AT, primary in vitro differentiated adipocytes (not shown) and primary preadipocytes, observing ample variability and difficulty in obtaining good data in the first two and the best results in the latter. We thus assessed ERK1/2 in preadipocytes isolated from visceral AT explants. As shown in Figure 5A, pre-exposure to U0126 effectively decreased cinacalcet- and spermine-induced pERK/ERK elevation in human primary preadipocytes. Figure 5B shows that U0126 abolished the increase in LC3II induced by spermine; however, it was unable to prevent the effect of cinacalcet, possibly implying the involvement of other pathways (Figure 5B).

### 2.4. Associations between AT CaSR, Autophagy and TNF-α mRNA

Based on our previous data [3], we evaluated whether the expression of TNF-α is associated with different autophagy markers in human AT explants. Figure S1 shows that AT TNF-α transcript content positively correlates with donors’ mRNA for the autophagy markers *atg5*, *lc3b* and *becn*. These observations led us to include TNF-α in the SEM model described below.

**Figure 6:**
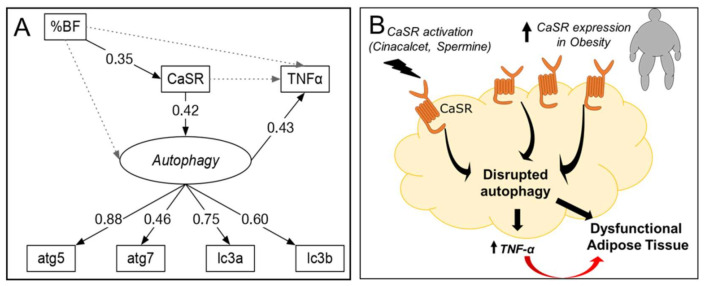
Standardized solution for SEM analysis and proposed model. (**A**) Solid and dashed arrows indicate the evaluated significant (*p* < 0.05) and non-significant associations, respectively. Numbers indicate significant standardized effects (from −1 to 1). Labels in italics represent latent variables (see methods section). %BF: body fat percentage. Variances for each variable can be found in Supplementary Materials (Table S5). (**B**) Proposed schematic model based on our findings.

The SEM analysis used a structurally recursive model (all proposed causalities are unidirectional, Figure 6) that is identified following criteria described in [26]: (1) one latent variable (*autophagy*) defined by four manifest variables (mRNA of atg5, atg7, lc3a and lc3b), and (2) 17 degrees of freedom, which result from 28 observations (7 observed variables) and 16 parameters. Supplementary Materials Tables S2–S5 provide details about the SEM fit, coefficients, residuals and variance for each variable, respectively. Figure 6 shows the standardized coefficients (number on each arrow) for the association between variables. These coefficients indicate the increase in units of standard deviation (SD) of one variable based on a dependent variable. For example, the model confirms the association between %BF and CaSR, indicating that an increase in one SD of %BF above its mean would increase CaSR by 0.35 SD above its mean, maintaining all other variables fixed. The SEM analysis supports a sequence in which %BF increases CaSR, which increases the latent variable *autophagy* and results in increased TNF-α mRNA. This explains 18% of the variance in TNF-α. (The residual variance of TNF-α not explained by the model is 82%, Table S5.) The model also indicates that CaSR correlates positively with *autophagy* (standardized coefficient of 0.42). Finally, the paths from %BF to autophagy and TNF-α are not significant (gray doted lines), supporting the hypothesis that CaSR and autophagy mediate the association between obesity (%BF) and expression of TNF-α in our model.

## 3. Discussion

The present work shows that subjects with greater levels of adiposity have higher content of CaSR transcripts in visceral AT. Interestingly, we found that greater CaSR mRNA also correlates with higher autophagy marker transcripts, and that CaSR activation induces LC3II accumulation in human AT explants. This LC3II response may represent defective autophagosome degradation, since the autophagy flux tends to decrease.

Previous studies have reported that CaSR modulates autophagy in THP-1 macrophages [20], cardiac fibroblasts [21] and kidney cells [22]. The association between CaSR and autophagy in AT has only been recently explored [3], and our present findings further contribute to understanding well-known autophagy dysregulation in AT of people with obesity.

Obesity frequently involves visceral fat accumulation, causing local and systemic inflammation [27]. In AT inflammation, higher CaSR activity is expected to further upregulate proinflammatory cytokines [16], likely enhancing inflammation-linked obesity disorders. Considering that CaSR mRNA shows consistency with protein expression [28,29], we assessed CaSR transcripts in AT from subjects with obesity. Adipocytes in vitro show elevated CaSR expression when exposed to proinflammatory cytokines or serum from donors with obesity [15]. Interestingly, we observed that CaSR mRNA in AT explants was positively correlated with %BF, which in addition to being an obesity indicator, is strongly associated with low-grade inflammation [30]. Together, these data fuel the hypothesis that CaSR could play an important role in perpetuating the inflammatory and dysfunctional state of AT.

Adipose tissue is composed of several cell types (adipocytes, adipocyte precursor cells (globally termed preadipocytes), cells of the immune system, fibroblasts, endothelial cells and stem cells), and alterations in many of them have been suggested to contribute to obesity-related tissue dysfunction. Each of these cells could be potential targets in our studies, provided they express functional CaSR. A recent review [31] concluded that the current literature offers disparate results regarding which cell type within AT determines overall tissue autophagy, or its autophagic response to nutritional stress or obesity-related metabolic derangements, highlighting that this is a pending matter in the field. We and others have shown the presence and activation of CaSR in preadipocytes and adipocytes from human and rodent adipose tissue [16,32]. Additionally, since circulating and sinovial human monocytes and macrophages have shown to express CaSR [33], with a proinflammatory response upon its activation [34], we may speculate that adipose-resident macrophages express CaSR as well, possibly with similar proinflammatory effects, but to our knowledge this has not been directly evaluated. The present study expands our previous work, which focused on autophagy in preadipocytes, since they are the main fat-specific cell with a proinflammatory potential [35]. In addition, interaction between different cell types, and with the extracellular matrix, is also involved in development of the dysfunctional phenotype [36], and our recent in vitro studies have suggested that CaSR may promote these deleterious interactions as well [37]. By confirming previous autophagy findings in whole visceral AT, we provide a physiological approach that involves actual interaction of all tissue components. An important next step would be to assess the relative contribution of the other cell types that express CaSR within AT explants.

We show that CaSR mRNA abundance is positively correlated with mRNA of the autophagy factors *atg5, atg7* and *lc3b* in AT. This is relevant considering that AT transcriptional regulation of autophagy in obesity is a potential AT dysfunction signature [23]. Our previous work showed that CaSR’s effects are mediated by nuclear factor kappa B (NF-κB) [16], a transcription factor that is also likely involved in AT dysregulation of autophagy [23].

It has been reported that *atg5* mRNA may be clinically relevant in obesity, since it explains about 50% of visceral adiposity and adipocyte hypertrophy [2]. Additionally, CaSR activation in visceral-derived adipose precursors increases adipogenesis [17]. Considering that increased autophagy is involved in adipogenesis and lipogenesis in AT [4,38], it is possible that in energy overload, CaSR and autophagy could concertedly mediate a deleterious visceral AT mass expansion.

Autophagy is modulated by the pharmacological CaSR allosteric activator cinacalcet in HeLa cells [39]. Spermine and other polyamines are less specific CaSR agonists with a potential physiological role in the context of obesity [40,41,42]. They have an established role modulating autophagy [43,44]; however, these studies did not explore the role of CaSR in mediating these effects. We observed that CaSR activation with cinacalcet or spermine elicited an increase of *atg7, becn* and *lc3a* mRNA transcripts, which is consistent with our previous report in human preadipocytes [3]. This is interesting, considering that AT explants from people with obesity represent a non-naive model comprising inflammation, fibrosis and oxidative stress, and suggests that CaSR’s effect occurs regardless of the pathophysiological state. On the other hand, we observed a null response in *atg5* and *lc3b* mRNA expression, which was unexpected given their well-reported role as markers of induced autophagy in AT in obesity condition [2]. This may be due to the high correlation of these factors with basal levels of CaSR mRNA, suggesting a relatively high basal expression, thus less inducible by CaSR agonists.

We show that cinacalcet and spermine induce LC3II expression during the first 2–6 h under conditions of flux inhibition (+CQ). Nevertheless, at 24 h of treatment in conditions without flux inhibition (−CQ), we observed a rise in LC3II (reaching +CQ levels in a case), which may be interpreted as CaSR-induced lower degradation of autophagosomes, resulting in their accumulation. We show that p62 protein increases with cinacalcet exposure, which could indicate a decrease in autophagy, since p62 is degraded when autophagy flux is active [25]. Moreover, autophagy flux analysis showed a trend towards a decrease, which could support this interpretation. This observation is in line with data reported by the Mizushima group describing cinacalcet as an inhibitor of late stages of autophagy in Hela cells [39]. Therefore, we propose that CaSR has a dual role of inducing autophagy initiation but impairing autophagosome-lysosome fusion and subsequent degradation of the cargo. A similar dual role modulating autophagy was reported for proinflammatory cytokines in pancreatic beta cells, where the early steps of autophagy are stimulated while blocking autophagic flux, resulting in an increase in ER stress and cell death [45]. This likely reflects CaSR-triggered impairment of the “cleaning” function of autophagy that is key to preserve cellular homeostasis, inducing an accumulation of non-degraded autophagosomes.

ERK1/2 activation was involved in CaSR-induced autophagy induction by spermine and not by cinacalcet in preadipocytes. This may reflect the well-described biased agonism of CaSR activation [11,12,46], which has been documented for polyamines toward ERK1/2 signaling in HEK293-CaSR cells [46]. Our previous reports showed that CaSR activation by cinacalcet triggers ERK1/2 pathway to regulate preadipocyte proliferation [13] and inflammasome activation [14]; however, this pathway probably does not mediate the effect on autophagy. The specific pathway involved in cinacalcet autophagy modulation remains to be elucidated. Unfortunately, given the limitation of reduced sample sizes of human visceral AT explants available, we were not able to investigate other cinacalcet- and/or spermine-induced second messengers and their related kinases.

We used an SEM analysis to test the hypothesis that CaSR mRNA in AT is associated with autophagy and that autophagy mediates CaSR-induced *TNF-α* expression. The link between autophagy, *TNF-α* expression and CaSR is consistent with our previous findings where CaSR-induced autophagy in preadipocytes mediated TNF-*α* secretion [3]. Our model indicates that CaSR could mediate the association between obesity and autophagy in AT that has been consistently documented [2,7,30,47]. These findings support the idea that even though autophagy and inflammation are regulated and necessary for cellular homeostasis, when dysregulated they become pathological. Thus, the role of CaSR in obesity may relate to both autophagy and inflammation.

This study is the first approach to explore the role of CaSR on whole human visceral AT autophagy. Our study confirms previous data in isolated preadipocytes, that CaSR activation increases the expression of the autophagy marker LC3II. However, given methodological limitations linked to human AT work, we could not confirm with a second technique whether and how autophagy flux is affected. Our cross-sectional analyzes do not shed light on timing or causal associations between obesity, CaSR expression and autophagy markers; however, visceral AT autophagy upregulation has been reported in patients with obesity before insulin resistance or T2D are evidenced, likely preceding obesity comorbidity [2]. By multivariate analysis, we show that elevated CaSR mRNA expression in subjects with higher %BF is a contributing factor to obesity-related elevation of autophagy at the transcriptional level. Autophagy modulation has been proposed in different organs as a potential target of disease treatment [48], and its cell tissue-specific regulation will be a particular challenge from the perspective of AT.

In summary, we show a complex dual regulation of CaSR in autophagy, increasing autophagy machinery (mRNA transcripts) but at the same time disturbing flux as evidenced by LC3II protein accumulation. This may reflect CaSR-triggered impairment of the “cleaning” function of autophagy that is key to preserve cellular homeostasis. These findings fuel the hypothesis thatCaSR could play an important role in perpetuating dysfunctional autophagy in AT from people with obesity.

## 4. Materials and Methods

### 4.1. Subjects and Visceral Adipose Tissue Experiments

Human visceral AT (greater omentum) was obtained from 60 subjects undergoing elective abdominal surgery. The protocol (FONDECYT Project #1150651) was approved by the Scientific Ethics Committee at the Institute of Nutrition and Food Technology, University of Chile on 14 August 2014. Each participant signed an informed consent. The anthropometric data were collected from clinical records before surgery (Table S6).

Visceral AT was obtained during surgery and processed within the first 2 h post-surgery. The tissue was washed several times with phosphate buffered saline (PBS, #02-023-5A, Biological Industries, Kibbutz Beit-Haemek, Israel) and minced into small pieces (2–3 mm^2^), and all visible connective tissue, blood clots and vessels were removed. Before the experiments, the explants were incubated for 24 h in medium 199 (M199, #M0393, Sigma-Aldrich, St Louis, MO, USA) and antibiotics (penicillin–streptomycin #03-031-1B, Biological Industries, Kibbutz Beit-Haemek, Israel) at 37 °C and 5% CO_2_, and the medium was changed three times in order to allow it to stabilize after processing. For each experimental condition, 100–150 mg AT was placed in 3 mL fresh M199. CaSR was activated using calcimimetic cinacalcet (#S1260, Selleck Chemicals, Houston, TX, USA) or orthosteric CaSR ligand spermine (#S4264, Sigma-Aldrich, St. Louis, MO, USA), which is found in circulation and is elevated under conditions of inflammation such as obesity [42]. The negative allosteric CaSR modulator calhex 321 (#SML0668, Sigma-Aldrich, St. Louis, MO, USA) was used to inhibit CaSR activation. Concentrations and exposure times for CaSR activators were based on our previous work [3,13]. To assess autophagy flux, we used the autophagosome degradation inhibitor chloroquine (CQ, #C6628, Sigma-Aldrich, St. Louis, MO, USA). CaSR activators were used at 2, 6, 16 or 24 h depending on the experiment, while calhex 231 was used 1 h before exposure to the CaSR activator.

### 4.2. Primary Preadipocyte Isolation and Culture

Isolation of human AT-derived (primary) preadipocytes was performed as described previously [17]. After passage 3 in culture with DMEM:F12 and 10% FBS, preadipocytes were switched to 2.5% FBS and exposed to cinacalcet, spermine and/or the upstream ERK (MAPK) inhibitor U0126 (#U120, Sigma-Aldrich, St Louis, MO, USA), plus the autophagy flux inhibitor CQ for the last 2–3 h. CaSR activators were used for 6 h, while U0126 was used 1 h before exposure to the CaSR activator.

### 4.3. RNA Isolation, Reverse Transcription and mRNA Expression by RT-PCR

Whole AT explants were homogenized with TRIzol^®^ (#15596018 Invitrogen, CA, USA) and total RNA was isolated using a PureLinkRNA mini kit (#12183018A, Invitrogen CA, USA) according to the manufacturer’s instructions. For cDNA synthesis, reverse transcription was performed using a High-Capacity cDNA Reverse Transcription Kit (#4368813, Applied Biosystems, Foster City, CA, USA). To assess mRNA abundance, the SYBRFAST qPCR kit (#4385612, Applied Biosystems, CA, USA) was used with conditions of 20 s preincubation at 95 °C, followed by 40 cycles at 95 °C for 3 s and 60 °C for 30 s. The relative mRNA expression was calculated based on Pfaffl [49], normalized by the reference gene GAPDH and expressed as a fold change from the respective control. The primer sequences (PCR) are shown in Table S7.

### 4.4. Protein Abundance by Western Blot

AT was homogenized using RIPA lysis buffer (150 mmol/L NaCl, 10 mmol/L Tris base, 1% deoxycholic acid, 4.5 mM EDTA and 1% Triton), supplemented with 1 mmol/L sodium orthovanadate (#S6508, Sigma-Aldrich, St. Louis, MO, USA), 1.5 μmol/L pepstatin A (#P5318, Sigma-Aldrich, St. Louis, MO, USA), PhosStop phosphatase inhibitor cocktail (# 4906845001, Roche, Basel, Switzerland) and C*O*mplete^®^ protease inhibitor cocktail (# 11836153001, Roche, Basel, Switzerland). The lysate was centrifuged (12,000× *g*, 15 min), and the protein concentration was determined (bicinchoninic acid-based assay, #23277, Pierce, Rockford, IL, USA). The lysates were adjusted to 1 μg/μL protein in SDS-Page loading buffer (240 mmol/L Tris-HCl, pH 6.8, 8% SDS, 40% glycerol and 20% 2-mercaptoethanol) and heat-denatured at 100 °C for 5 min. Twenty to thirty μg protein were loaded on a polyacrylamide gel and electrophoresed at 80–120 V for 1.5 h. Depending on their size, the proteins were electrotransferred to nitrocellulose 0.22 µm or polyvinylidene difluoride (PVDF) 0.45 µm membranes at 90 V for 1–1.5 h in a buffer containing 24 mmol/L Tris, 194 mmol/L glycine and 20% methanol. The membranes were then blocked with 5% non-fat milk or BSA in Tris-buffer saline (TBS) and incubated with primary antibodies (LC3B Cell Signaling #2775, 1:500; Atg7 Cell Signaling #2631; Beclin-1 Cell Signaling #3738; β-actin Santa Cruz #sc-47778, 1:3000, ERK Santa Cruz #sc-94, 1:1000 and pERK Santa Cruz #sc-7383, 1:500) resuspended in TBS with 0.05% tween 20 (#7949, Sigma, St. Louis, MO, USA) and 5% BSA. Immune complexes were detected using peroxidase-conjugate secondary antibodies (Goat anti-Rabbit #sc-2004 and Goat-anti Mouse #sc-2005, Santa Cruz Biotechnology, Santa Cruz, CA) and the enzyme substrate ECL (# 20-500-500A and 20-500-500B, Biological Industries, Cromwell, CT, USA). Images were scanned in C-DiGit Blot Scanner (LI-COR Biosciences, Lincoln, NE, USA) and analyzed with ImageJ software (NIH, USA).

### 4.5. Statistical Analysis

To compare control vs. treated tissue, variables were evaluated using the non-parametric Wilcoxon signed rank test. Two-way ANOVA and Holm–Sidak’s multiple comparison and post-hoc test were used when appropriate. For all variables, normal distribution was tested and data not normally distributed were log transformed. The associations were analyzed by Pearson’s correlation test. We performed sensitivity analyzes of correlations by excluding extreme points that may “force” a significant association. Data are shown as means ± standard error and individual experiment values. The *p* values less than 0.05 were considered statistically significant.

Structural equation modeling (SEM) analysis evaluated associations between body fat percentage (%BF), mRNA for CaSR, TNF-α and autophagy markers from the AT donor. This approach is a multivariate technique that simultaneously examines relationships between different variables [50]. SEM analysis is broadly applied in social sciences and also has been used for multivariate analysis in biomedicine [51]. An advantage of SEM is that it can use latent variables, composed of two or more single variables, and reflect a concept that cannot be represented by a single measurement. The original dataset for SEM analysis included 60 subjects (43 female and 17 male) with 6.1% missing data. Eliminating subjects with at least two missing data points from gene mRNA expression reduced the subjects to 49 (n = 33 female) with 2.4% missing data. Each variable was examined for normality using quantile-quantile (Q–Q) plots and transformed using a Box–Cox transformation of original data plus one unit per variable. Missing data were imputed using the R mice package with default settings [52]. SEM analysis was conducted using the R package lavaan 0.6–5 [53] using maximum likelihood. An SEM model fit was evaluated using different tests (see Supplementary Table S2).

## Supplementary Materials

Supplementary materials can be found at https://www.mdpi.com/1422-0067/21/20/7617/s1.
