# Supplementary material for: Calcium-Sensing Receptor in Adipose Tissue: Possible Association with Obesity-Related Elevated Autophagy
Source: Int J Mol Sci. 2020 Oct 15;21(20):7617. doi: 10.3390/ijms21207617 (PMC7590196; doi:10.3390/ijms21207617)
Supplement: Supplementary file 1 [file ijms-21-07617-s001.pdf]

SUPPLEMENTARY INFORMATION

Calcium sensing receptor in adipose tissue: possible association with obesity-related elevated autophagy

Pamela Mattar[1], Sofía Sanhueza[1], Gabriela Yuri[1], Lautaro Briones[1,2], Claudio Perez-Leighton[3], Assaf Rudich[4,5], Sergio Lavandero [6,7,8], Mariana Cifuentes[1,6,7,*]

1. *Instituto de Nutrición y Tecnología de los Alimentos (INTA), Universidad de Chile, Santiago 7830490, Chile.*
2. *Facultad de Ciencias de la Salud, Departamento de Nutrición y Salud Pública, Universidad del Bío-Bío, Andrés Bello 720, Chillán 3800708, Chile.*
3. *Facultad de Ciencias Biológicas, Departamento de Fisiología, Pontificia Universidad Católica de Chile, Santiago 3580000, Chile.*
4. *Department of Clinical Biochemistry and Pharmacology, Ben-Gurion University of the Negev, 84103, Beer-Sheva, Israel.*
5. *The National Institute of Biotechnology in the Negev, Ben-Gurion University of the Negev, 84103, Beer-Sheva, Israel.*
6. *Advanced Center for Chronic Diseases (ACCDiS), Facultad de Ciencias Químicas y Farmacéuticas & Facultad de Medicina, Universidad de Chile, Santiago 8380492, Chile.*
7. *Center for Exercise, Metabolism and Cancer (CEMC), Facultad de Medicina, Universidad de Chile, Santiago 8380492, Chile.*
8. *Cardiology Division, Department of Internal Medicine, University of Texas Southwestern Medical Center, Dallas, Texas 75390, USA.*

* Correspondence Dr. Mariana Cifuentes, Address: El Libano 5524, Macul, Santiago *7830490*, Chile. Electronic address: mcifuentes@inta.uchile.cl, Telephone: (56) 229781428

SUPPLEMENTARY FIGURES

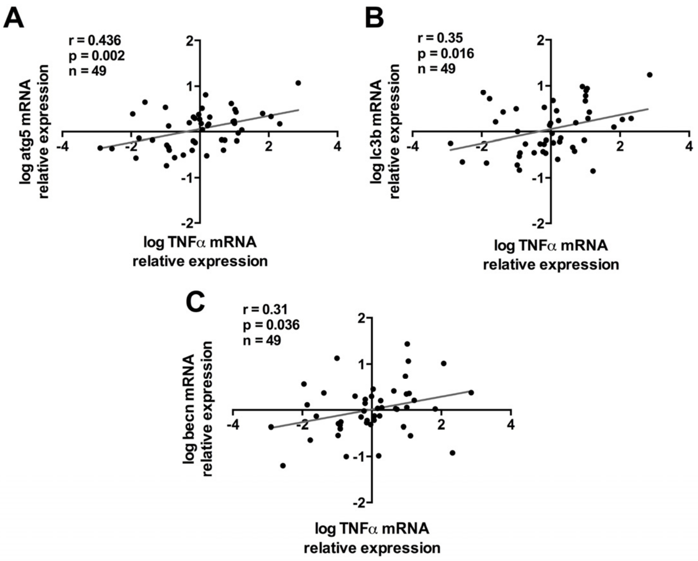

Figure S1. Adipose tissue *TNF-α* mRNA content positively correlates with donors' mRNA of autophagy markers atg5, lc3b and becn. All values expressed as log, Pearson correlation coefficient was calculated for the association between *TNF-α* mRNA and (A) atg5, (B) *lc3b and (c) becn*. Each graph depicts the r, and p values, as well as the number of independent donor explants analyzed.

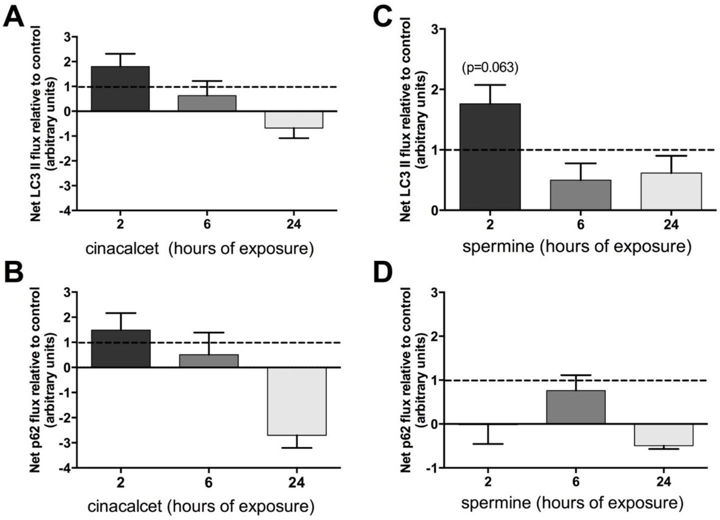

Figure S2. Net flux protein expression for LC3II or p62 was determined after 2, 6 and 24 h of 2µM cinacalcet (A and B) or 100 µM spermine (C and D). LC3 and p62 abundance was normalized by β-actin and showed in representative images in Fig. 3. Differences between vehicle (1) and each treated condition were determined by Wilcoxon signed rank test. p value is presented in each bar. The flux was determined by subtraction of +CQ and -CQ condition in each time of treatment. n=4 (A, B and D) and n=6 (C) independent experiments.

SUPPLEMENTARY TABLES

Table S1. Anthropometric and biochemical characteristics of adipose tissue donors in the SEM analysis

Variables	median (p25-p75)
**Subjects [M/F]**	60 [17/43]
**Age (years)**	40.2 (33.8-47.0)
**BMI (kg/m^2)**	33.0 (28.9-35.0)
**WC (cm)**	102.7 (90.8-112.1)
**BF (%)**	42.6 (35.6-45.3)

M: Male, F: Female, BMI: Body Mass Index, WC: Waist circumference, BF: Body Fat.

Table S2. Primer sequences for qPCR

mRNA target	Access	Forward primer (5′→ 3′)	Reverse primer (5′→ 3′)
**CaSR**	NM_001178065	GATGAGACAGATGCCAGTGC	AAAGAGGGTGAGTGCGATCC
**atg5**	NM_001286106.1	AACTGAAAGGGAAGCAGAACCA	CCATTTCAGTGGTGTGCCTTC
**atg7**	NM_006395.2	CGTTGCCCACAGCATCATCTTC	CACTGAGGTTCACCATCCTTGG
**becn**	NM_003766.4	GGCTGAGAGACTGGATCAGG	CTGCGTCTGGGCATAACG
**lc3a**	NM_032514.3	CCAGCAAAATCCCGGTGAT	TGGTCCGGGACCAAAAACT
**lc3b**	NM_022818.4	ACCATGCCGTCGGAGAAG	GGTTGGATGCTGCTCTCGAA
**GAPDH**	NM_002046	GAAGGTGAAGGTCGGAGTCAAC	CAGAGTTAAAAGCAGCCCTGGT
**TNF-α**	NM_000594.2	CCAGGCAGTCAGATCATCTTCTC	AGCTGGTTATCTCTCAGCTCCAC

Table S3. Pearson's correlation coefficient for the association between mRNA CaSR versus anthropometric variables

	n	r	p
**BMI**	49	0.256	0.076
**%BF**	47	0.458	**0.001**
**WC**	42	0.166	0.292

BMI: Body Mass Index, BF: Body Fat, WC: Waist circumference

Table S4. FIT of SEM model

Test	value
**X^2**	11.612(11) = 0.39
**CFI**	0.99
**LTI**	0.98
**RMSEA**	0.034[0, 0.156] = 0.501
**SRMR**	0.06

X^2: chi-square, CFI: comparative fit index, LTI: Tucker-Lewis index, RMSEA: root mean square error of approximation, and SRMR: standardized root mean square residual.

Table S5. Coefficients of SEM model

Latent Variables	non-std coeff. ± standard error	p value	Std. coeff.
Autophagy			
**~ atg5**	1 ± 0	NA	0.88
**~ atg7**	0.72 + 0.22	0.001	0.46
**~ lc3a**	1.4 + 0.24	0.0000	0.752
**~ lc3b**	1.34 + 0.31	0.0000	0.597
**Regressions**	**non-std coeff. ± standard error**	**p value**	**Std. coeff.**
***Autophagy* ~ %BF**	0.01 + 0.04	0.7731	0.043
**TNF-α ~ %BF**	0.04 + 0.12	0.7347	0.041
**CaSR ~ %BF**	0.54 + 0.18	0.0032	0.350
**TNF-α ~ *Autophagy***	1.46 + 0.59	0.0134	0.428
***Autophagy* ~ CaSR**	0.08 + 0.03	0.0040	0.417

Note: ~ means explain for

Table S6. Residuals of SEM model

	atg5	atg7	lc3b	lc3a	TNF-α	CaSR	%BF
**atg5**	0.000	0.000	0.000	0.000	0.001	-0.001	0.000
**atg7**	0.000	0.000	-0.001	0.003	0.002	0.007	0.002
**lc3b**	0.000	-0.001	0.000	0.000	0.000	0.003	-0.001
**lc3a**	0.000	0.003	0.000	0.000	-0.011	-0.004	-0.002
**TNF-α**	0.001	0.002	0.000	-0.011	0.000	0.000	0.000
**CaSR**	-0.001	0.007	0.003	-0.004	0.000	0.000	0.000
**%BF**	0.000	0.002	-0.001	-0.002	0.000	0.000	0.000

Table S7. Variances of SEM model

Variable	non-std coeff. ± standard error	p value	Std. coeff.
**atg5**	0.002 ± 0.001	0.034	0.23
**atg7**	0.013 ± 0.002	0.000	0.79
**lc3b**	0.01 ± 0.003	0.000	0.43
**lc3a**	0.022 ± 0.005	0.000	0.64
**TNF-α**	0.065 ± 0.013	0.000	0.82
**CaSR**	0.163 ± 0.022	0.000	0.88
Autophagy	0.006 ± 0.002	0.000	0.81
